# Filamentous actin binding enables βCaMKII to regulate bidirectional plasticity in cerebellar Purkinje cells

**DOI:** 10.1186/1471-2202-14-S1-P375

**Published:** 2013-07-08

**Authors:** Thiago M Pinto, Maria J Schilstra, Volker Steuber

**Affiliations:** 1 Science and Technology Research Institute, University of Hertfordshire, Hatfield, Herts, AL10 9AB, UK

## 

Synaptic plasticity is an activity-dependent alteration in the strength of synaptic connections between pre and postsynaptic neurons. The long-term strengthening and weakening of synapses are known as long-term potentiation (LTP) and long-term depression (LTD), respectively. Long-lasting changes in synaptic strength are thought to be the basis of learning and the formation of memories. In particular, these calcium-dependent forms of plasticity are involved in learning and pattern recognition by cerebellar Purkinje cells (PCs). Different levels of intracellular calcium mediate the induction of LTD and LTP between parallel fibres (PF) and PCs. The coincident activation of PF and climbing fibre (CF) input evokes large concentrations of intracellular calcium, which in turn lead to LTD. In contrast, the induction of LTP is induced by small increases in intracellular calcium concentration in response to PF input alone.

Research has pointed out that complex interactions between many intracellular signalling components underlie cerebellar plasticity. In particular, calcium/calmodulin-dependent protein kinase II (CaMKII) and protein phosphatase 2B (PP2B) mediate the phosphorylation and dephosphorylation of AMPA receptors, regulating LTD and LTP in cerebellar PCs. The CaMKII holoenzyme comprises different isoforms such as αCaMKII and βCaMKII. Although βCaMKII is the predominant isoform of CaMKII in the cerebellum, its role in cerebellar learning and memory has yet to be established.

Recent experiments with Camk2b knockout mice, which lack the β isoform of CaMKII, have addressed the role of βCaMKII in plasticity in cerebellar PCs. These studies have revealed that βCaMKII regulates the direction of plasticity at PF-PC synapses [1]. Experimental protocols that induce LTP in wild-type mice, which contain both α and βCaMKII isoforms, result in LTP in knockout mice that lack βCaMKII, and vice versa. However, the underlying mechanism that may explain these experimental findings is not clear. Van Woerden et al. [1] have suggested that the binding of CaMKII to F-actin could underlie the switch of direction of synaptic plasticity. The βCaMKII, but not αCaMKII, isoform can bind to F-actin, which could result in clustering of the CaMKII holoenzyme to F-actin, making it unavailable for phosphorylation of AMPA receptors.

To investigate the role of βCaMKII in the regulation of bidirectional plasticity at PF-PC synapses, we developed a simple kinetic model of the phosphorylation and dephosphorylation of AMPA receptors by CaMKII and PP2B. The model is based on our recent model of CaMKII activation by calcium/calmodulin [2,3]. We then included the binding of F-actin to CaMKII to simulate the induction of plasticity in wild-type mice, while in the knockout mice that lack βCaMKII F-actin binding was omitted.

Our simulation results replicate the experimental observations by van Woerden et al. [1] and unravel how the βCaMKII isoform can control the sign reversal of plasticity at PF-PC synapses. We demonstrate that the binding of F-actin to βCaMKII can indeed contribute to the control of bidirectional plasticity at these synapses. At low concentrations of calcium in response to PF stimulation alone, the loss of F-actin binding in the knockout mice enhances the availability of active CaMKII when compared to the wild-type mice. This mechanism leads to the induction of LTD instead of LTP. However, for the large increases of calcium concentrations that result from coincident activation of PF and CF, the reduction in the CaMKII concentration in the knockout mice leads to AMPA receptor dephosphorylation by PP2B, favouring the induction of LTP rather than LTD.
